# Rapamycin Modulates the Polarisation of CD4+ T Cells Towards T_H_1 Cells in Patients with Active Granulomatosis with Polyangiitis and Microscopic Polyangiitis

**DOI:** 10.3390/jcm14248720

**Published:** 2025-12-09

**Authors:** Jang Woo Ha, Taejun Yoon, Oh Chan Kwon, Yong-Beom Park, Sang-Won Lee

**Affiliations:** 1 Division of Rheumatology, Department of Internal Medicine, Yongin Severance Hospital, Yonsei University College of Medicine, Yongin 16995, Republic of Korea; hjwnmk@yuhs.ac; 2 Division of Rheumatology, Department of Internal Medicine, Yonsei University College of Medicine, Seoul 03722, Republic of Korea; tjyoonn92@gmail.com (T.Y.);; 3 Division of Rheumatology, Department of Internal Medicine, Gangnam Severance Hospital, Yonsei University College of Medicine, Seoul 06273, Republic of Korea; 4 Institute for Immunology and Immunological Diseases, Yonsei University College of Medicine, Seoul 03722, Republic of Korea

**Keywords:** rapamycin, microscopic polyangiitis, granulomatosis with polyangiitis, helper T cells, polarisation

## Abstract

**Objective:** This study investigated whether rapamycin could modulate the polarisation of CD4+ T cells towards T_H_1, T_H_2, T_H_17, and Treg cells using peripheral blood mononuclear cell (PBMC) obtained from patients with granulomatosis with polyangiitis and microscopic polyangiitis (GPA/MPA). **Methods:** Twenty patients with GPA/MPA were included in this study. Their stored PBMCs were cultured and stimulated with anti-CD3 and anti-CD28 antibodies for 72 h in the presence or absence of rapamycin (10 nM). The cells were stained for surface markers with anti-CD4-FITC and anti-CD25-APC, followed by intracellular staining using anti-interferon (IFN)-γ-PE, anti-IL-4-PerCP-Cy5, anti-IL17A-APC, and anti-Foxp3-PE. The stained cells were analysed using a flow cytometer. **Results:** The median age of the 20 GPA/MPA patients (10 men and 10 women) was 65.5 years. Rapamycin treatment significantly modulated the polarisation of CD4+IFN-γ+ T (T_H_1) cells compared to no treatment among GPA/MPA patients. In addition, the polarisation of CD4+IFN-γ+ T (T_H_1) cells was also significantly reduced in rapamycin-treated PBMC obtained from active patients compared to untreated PBMC from the same patients; however, these alterations were not observed in inactive patients. Conversely, rapamycin treatment did not affect the polarisation of CD4+IL-4+ T (T_H_2), CD4+IL-17+ T (T_H_17), or CD4+FoxP3+CD25+ T (Treg) cells, regardless of GPA/MPA activity. **Conclusions:** This study was the first pilot study to demonstrate that rapamycin modulates the polarisation of CD4+ T cells towards CD4+IFN-γ+ T cells in active GPA/MPA.

## 1. Introduction

The mammalian target of rapamycin (mTOR) is a conserved serine/threonine protein kinase and a member of the phosphoinositide 3-kinase (PI3K) family. mTOR regulates cell growth, division, differentiation, metabolism, survival, and death by intracellular signal transduction initiated by diverse extracellular stimuli and transferred via the PI3K-protein kinase B pathway [1]. mTOR exists in two complex forms: mTOR complex 1 (mTORC1) and mTORC2. mTORC1 is sensitive to rapamycin and is primarily involved in cell growth and metabolism. Meanwhile, mTORC2 is relatively less sensitive to rapamycin and participates in cell survival and division [2]. In terms of T helper (T_H_) cells, mTOR plays a critical role in T cell differentiation and function. In particular, mTORC1 induces and promotes the differentiation of T_H_1 cells together with interleukin (IL)-12, and that of T_H_17 cells alongside transforming growth factor-β and IL-6 [2].

Granulomatosis with polyangiitis (GPA) and microscopic polyangiitis (MPA) are subtypes of antineutrophil cytoplasmic antibody (ANCA)-associated vasculitis (AAV), which is histopathologically characterised by fibrinoid necrotising vasculitis with no or few immune complexes in small vessels [3]. In terms of T cell immunology, the relatively dominant T_H_1 cell-mediated immune responses and the polarisation towards T_H_17 cells and impaired Treg cell function are major features of the pathogenesis of GPA/MPA [4,5]. Therefore, we can infer that rapamycin alters and modulates CD4+ T cell subset populations, leading to the alleviation of GPA/MPA activity. However, no studies have assessed the role of rapamycin in altering T cell subset populations in GPA/MPA patients. Hence, to estimate the clinical significance of rapamycin in GPA/MPA, this study used peripheral blood mononuclear cells (PBMC) obtained from GPA/MPA patients [6] and investigated whether rapamycin can modulate the polarisation of CD4+ T cells towards T_H_1, T_H_2, T_H_17, and Treg cells.

## 2. Materials and Methods

### 2.1. Patients

This study retrospectively reviewed the medical records of 20 patients with AAV (4 GPA and 16 MPA patients) arbitrarily selected from the Severance Hospital ANCA-associated Vasculitides (SHAVE) cohort, an observational cohort of Korean patients with AAV. The inclusion criteria for the SHAVE cohort were as follows: (i) classification of GPA/MPA according to the 2007 European Medicine Agency algorithm, and the revised 2012 Chapel Hill Consensus Conference for vasculitides nomenclature [3,7], (ii) fulfilment of the 2022 new classification criteria for GPA/MPA [8,9], (iii) first classification of GPA/MPA at this hospital; (iv) sufficient medical records collected at diagnosis, and (v) consent for blood collection. This study was approved by the Institutional Review Board (IRB) of Severance Hospital, Seoul, Republic of Korea (IRB number 4-2016-0901, approval date 11 November 2016). Written informed consent was obtained from patients at the time of blood sampling. The IRB waived the requirement of written informed consent when it had been previously obtained at the time of enrolment in the SHAVE cohort.

### 2.2. Clinical Data at Blood Sampling (at Diagnosis)

Age and sex were collected as demographic data. AAV subtypes, ANCA type and positivity, the Birmingham Vasculitis Activity Score (BVAS), and the Korean version of the Short-Form 36-Item Health Survey Physical and Mental Component Summaries (SF-36 PCS and MCS) were collected as AAV-specific indices [10,11]. Epidemiological data, including age and sex, and laboratory results, including erythrocyte sedimentation rate and C-reactive protein levels, were also recorded.

### 2.3. Definition of Active AAV

In this study, we tentatively defined active AAV as BVAS > 5, as it was performed and described in our previous study [6].

### 2.4. PBMC Isolation and Storage (at Diagnosis)

Whole blood was obtained from GPA/MPA patients on consent and collected in EDTA tubes. PBMCs were isolated from the EDTA tube samples by Ficoll density-gradient centrifugation, stored at −80 °C in a freezing isopropanol container for 24 h, and transferred into the vapour phase of a liquid nitrogen tank.

### 2.5. PBMC Culture and Rapamycin Treatment

Stored PBMCs from 10 patients with AAV exhibiting high disease activity (BVAS  > 5) and 10 patients with low disease activity (BVAS  ≤ 5) were cultured and stimulated with anti-CD3 and anti-CD28 antibodies for 72 h, in the presence or absence of rapamycin (10 nM) [12].

### 2.6. Fluorescence-Activated Cell Sorting (FACS)

Prior to FACS staining, the cells were restimulated with phorbol 12-myristate 13-acetate, ionomycin, and Golgi inhibitors for 5 h. Subsequently, the cells were stained for surface markers with anti-CD4-FITC and anti-CD25-APC (BioLegend, San Diego, CA, USA), followed by intracellular staining using anti-IFN-γ-PE (BioLegend), anti-IL-4-PerCP-Cy5 (BD Biosciences, Oxford, UK), anti-IL-17A-APC (eBioscience, San Diego, CA, USA), and anti-Foxp3-PE (Invitrogen, Carlsbad, CA, USA). The stained cells were analysed using a FACSVerse flow cytometer and FlowJo v10 software.

### 2.7. Statistical Analyses

All statistical analyses were performed using SPSS version 26 (IBM Corporation, Armonk, NY, USA) for Windows (Microsoft Corporation, Redmond, WA, USA). Continuous and categorical variables were expressed as medians (interquartile ranges [IQR]) and numbers (percentages). Significant differences between the two categorical variables were analysed using the Chi-square and Fisher’s exact tests. Significant differences between two continuous variables were compared using the Mann–Whitney U test. *p*-value < 0.05 was considered to be statistically significant.

## 3. Results

### 3.1. Patients’ Characteristics and Comparison Between Patients with Active Disease and Those with Inactive Disease

The median age of the 20 GPA/MPA patients (10 men and 10 women) was 65.5 years. MPO-ANCA (or P-ANCA) and PR3-ANCA (or C-ANCA) were detected in 17 and 3 patients, respectively. The median BVAS, SF-36 PCS, and SF-36 MCS were 8.0, 52.5, and 49.3, respectively. When comparing variables based on GPA/MPA activity, active patients were older than inactive patients (71.0 vs. 57.5 years, *p* = 0.023). Additionally, active patients exhibited significantly higher serum creatinine levels than inactive patients (1.7 mg/dL vs. 0.7 mg/dL, *p* < 0.001) (Table 1). On the other hand, the clinical phenotypes and target organs of the 20 patients with AAV are presented in Supplementary Table S1.

### 3.2. Comparison of the Populations of T Cell Subsets According to Rapamycin Treatment

**Figure 1:**
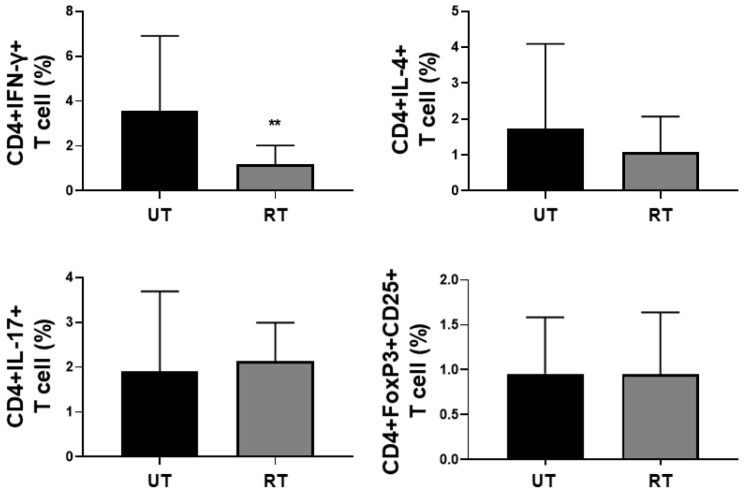
Populations of T cell subsets according to rapamycin treatment (UT N = 20, RT N = 20). IFN: interferon; UT: untreated PBMC; RT: rapamycin-treated PBMC; PBMC: peripheral blood mononuclear cell. ** *p* < 0.01.

Among CD4+ T cell subsets, rapamycin-treated PBMC exhibited a significantly reduced population of CD4+IFN-γ+ T (T_H_1) cells compared to untreated PBMC. However, no significant differences in the populations of CD4+IL-4+ T (T_H_2) cells, CD4+IL-17+ T (T_H_17) cells, or CD4+FoxP3+CD25+ T (Treg) cells were observed between rapamycin-treated and untreated PBMC (Figure 1). Therefore, we tentatively conclude that rapamycin treatment may modulate the polarisation of (naive) CD4+ T cells towards T_H_1 cells in PBMCs obtained from GPA/MPA patients.

### 3.3. Comparison of the Populations of T Cell Subsets According to Rapamycin Treatment and GPA/MPA Activity

**Figure 2:**
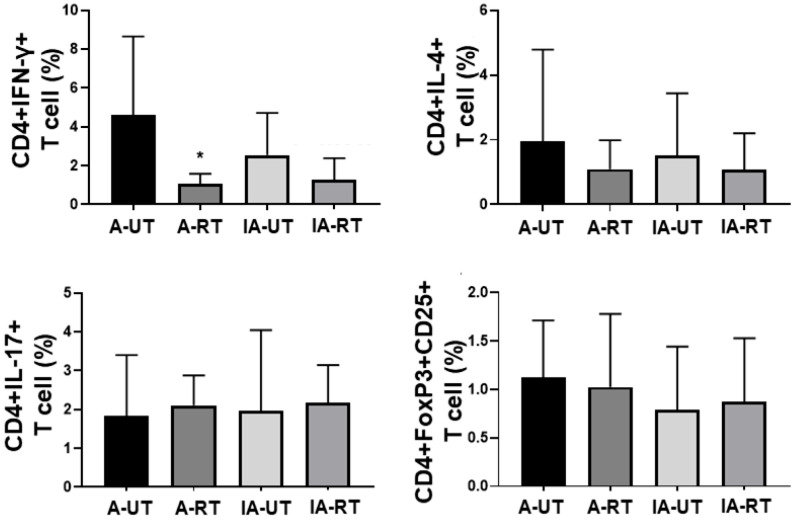
Populations of T cell subsets according to rapamycin treatment and GPA/MPA activity (A-UT N = 10, A-RT N = 10, IA-UT N = 10, IA-RT N = 10). GPA: granulomatosis with polyangiitis; MPA: microscopic polyangiitis; IFN: interferon; A-UT: untreated PBMC obtained from active GPA/MPA patients; A-RT: rapamycin-treated PBMC obtained from active GPA/MPA patients; IA-UT: untreated PBMC obtained from inactive GPA/MPA patients; IA-RT: rapamycin-treated PBMC obtained from inactive GPA/MPA patients; PBMC: peripheral blood mononuclear cell. * *p* < 0.05.

Among CD4+ T cell subsets, rapamycin-treated PBMC obtained from active GPA/MPA patients exhibited a significantly lower population of CD4+IFN-γ+ T cells than untreated PBMC obtained from the same patients, which was similar to the results of Figure 1. Meanwhile, among PBMC obtained from inactive GPA/MPA patients, the population of CD4+IFN-γ+ T cells in rapamycin-treated PBMC tended to be decreased compared to untreated PBMC; however, no statistically significant difference was found. The remaining T cell subset populations did not show significant differences in PBMC regardless of rapamycin treatment or AAV activity (Figure 2). Therefore, we tentatively conclude that rapamycin treatment may modulate the polarisation of (naive) CD4+ T cells towards T_H_1 cells in PBMCs obtained from active GPA/MPA patients but not from inactive patients.

## 4. Discussion

In this study, to estimate the clinical efficacy of rapamycin in GPA/MPA, we investigated whether rapamycin treatment can affect the polarisation of T cell subsets among PBMCs of GPA/MPA patients and obtained several findings. First, rapamycin treatment significantly modulated the polarisation of CD4+IFN-γ+ T cells compared to no treatment among GPA/MPA patients. Second, the polarisation of CD4+IFN-γ+ T cells was also significantly reduced in rapamycin-treated PBMC obtained from active patients compared to untreated PBMC from the same patients; however, these significant differences were found among inactive patients. Third, rapamycin treatment did not affect the polarisation of CD4+IL-4+ T cells, CD4+IL-17+ T cells, or CD4+FoxP3+CD25+ T cells, regardless of GPA/MPA activity. Therefore, based on these results, we conclude that among the T cell subsets of PBMCs obtained from GPA/MPA patients, rapamycin may significantly suppress the polarisation of CD4+ T cells towards CD4+IFN-γ+ T (T_H_1) cells with high vasculitic activity.

In addition to an increase in the population of CD4+IFN-γ+ T (T_H_1) cells, the population of CD4+IL-4+ T (T_H_2) cells tended to be decreased under rapamycin treatment, despite statistical significance. Based on these findings, we made the hypotheses on the effects of rapamycin on the polarisation of CD4+ T cells towards CD4+IFN-γ+ T (T_H_1) cells and CD4+IL-4+ T (T_H_2) cells in PBMCs of GPA/MPA patients. First, in terms of CD4+IFN-γ+ T cells, the ligands that stimulate their receptors may promote T_H_1 cell activation and differentiation through STAT4. In this process, mTORC1 can indirectly drive the role of STAT4 by interfering with the inhibitory effect of SOCS3 on STAT4 signalling. Rapamycin can block the inhibitory effect of mTORC1 on SOCS3, thereby enhancing the inhibitory effect of SOCS3 on STAT4 signalling. Accordingly, rapamycin can theoretically inhibit the activation and differentiation of CD4+IFN-γ+ T cells [2]. In this study, rapamycin treatment significantly suppressed the polarisation of CD4+IFN-γ+ T cells in PBMCs obtained from patients with active GPA/MPA, which was consistent with the above hypothesis (Figure 1 and Figure 2).

In terms of CD4+IL-4+ T (T_H_2) cells, when the ligands bind to their receptors, they may promote T_H_2 cell activation and differentiation through STAT6. In this process, mTORC1 can directly inhibit STAT6 signalling. Conversely, mTORC2 can indirectly increase the role of STAT6 by interfering with the inhibitory effect of SOCS5 on STAT6 signalling. Rapamycin can promote STAT6 signalling by directly having a negative influence on mTORC1. Accordingly, rapamycin can theoretically enhance the activation and differentiation of CD4+IL-4+ T cells. By contrast, rapamycin can switch off the inhibitory effect of mTORC2 on SOCS5, thereby accelerating the inhibitory effect of SOCS5 on STAT6 signalling. Accordingly, rapamycin can theoretically reduce the activation and differentiation of CD4+IL-4+ T cells [2]. In this study, despite no statistical significance, rapamycin treatment tended to suppress the polarisation of CD4+IFN-γ+ T cells in PBMCs obtained from patients with active GPA/MPA (Figure 1 and Figure 2). We inferred that in the pathogenesis of GPA/MPA, rapamycin may predominantly affect mTORC2 compared to mTORC1 in CD4+IL-4+ T cells and thus may suppress CD4+IL-4+ T cell activation and differentiation by interfering with the inhibitory effect of SOCS5 on STAT6 signalling.

In terms of CD4+IL-17+ T (T_H_17) and CD4+FoxP3+CD25+ T (Treg) cells, an increase in T_H_17 cells and a decrease in Treg cells were expected. This is because rapamycin can block the inhibitory effect of mTORC1 on SOCS3, thereby enhancing the inhibitory effect of SOCS3 on STAT3 signalling, and can directly block the inhibitory effect of mTORC1 on SMAD3 and SMAD4 signalling, resulting in a decrease in CD4+IL-17+ T cells and an increase in CD4+FoxP3+CD25+ T cells [2,13]. However, in this study, no significant alteration in the polarisation of CD4+IL-17+ T cells and CD4+FoxP3+CD25+ T cells under rapamycin treatment, regardless of activity (Figure 1 and Figure 2). Based on these results, we inferred that rapamycin may selectively act on mTORC1 and mTORC2 of T_H_1 and T_H_2 T cells but not T_H_17 and Treg cells.

On the other hand, to investigate the alterations in T_H_1 cell populations in response to rapamycin according to ANCA type and positivity, and AAV subtype. We divided patients into two groups according to MPO-ANCA positivity and PR3-ANCA positivity and compared the populations of T_H_1 cells in both UT and RT. When based on MPO-ANCA positivity, no differences in T_H_1 cell populations in both UT and RT were observed. When based on PR3-ANCA positivity, there were no gaps in T_H_1 cell populations, either. Also, when patients were divided into two groups according to AAV subtype, MPA and GPA, and compared, T_H_1 cell populations in UT as well as RT turned out not to differ significantly. Additionally, we evaluated the correlation of T_H_1 cell populations in UT and RT with AAV-specific indices and acute-phase reactants; however, no statistically significant correlations among them were found. Therefore, in this study, we could infer that the extent of response of T_H_1 cells to rapamycin may not be remarkably associated with ANCA type, AAV subtype, disease activity, and inflammatory burden.

Additionally, in this study, active patients exhibited a significantly higher age at diagnosis than inactive patients (71.0 years vs. 57.5 years). Given that the T_H_1 cell population/differentiation and response are more intact and efficient in young patients compared to old patients [14], at first, it could be inferred that relatively younger inactive patients would have exhibited a higher T_H_1 cell population and a subsequently higher T_H_1 cell response amplitude to rapamycin. Conversely, however, the results regarding T_H_1 cell patterns were actually more pronounced in the group of older active patients compared to the opposite group. Based on these results and the age-related (or age-dependent) T_H_1 cell population/differentiation and response, it was believed that the significant difference in age at diagnosis might not have negatively affected the major results of this study. Instead, this implied that AAV activity primarily contributed to T_H_1 cell population/differentiation and its response to rapamycin.

The strength of this study is that it is the first pilot study to demonstrate that rapamycin significantly modulates the polarisation of CD4+IFN-γ+ T (T_H_1) cells among T cell subsets in PBMC obtained from patients with active GPA/MPA. Meanwhile, this study has several limitations. The first limitation is that the number of patients donating PBMCs was not sufficiently large. The significantly older age in the active group and the absence of healthy controls included in this study may also represent an additional limitation of this study. The second critical limitation is the lack of additional information on the mTORC1/mTORC2-related intracellular signalling pathways of CD4+ T cells. We acknowledge that direct assessment of mTOR pathway activity, phenotypic alteration in T cell subsets, and further, phosphorylated or total phosphatidylinositol 3-kinases levels in patient-derived T cells would be essential to substantiate our interpretation; however, due to the limitations of this study as a pilot study, we plan to evaluate these issues in future work. Finally, the role of mTOR in the pathogenesis of AAV has not been clearly elucidated; however, the association between mTOR and the CD4+ T cell subsets was demonstrated. Therefore, this study indirectly investigated the role of mTOR in the pathogenesis of AAV by unveiling the relationship between mTOR and AAV through rapamycin, but could not prove a direct role of mTOR in AAV pathogenesis.

Nevertheless, we believe that this study has clinical significance as a pilot study in that it can serve as a cornerstone for further research. Further in vivo studies using animal models for autoimmune experimental vasculitis will provide more reliable and dynamic therapeutic potential of rapamycin in patients with GPA/MPA.

## 5. Conclusions

To our knowledge, this study was the first pilot study to demonstrate that rapamycin modulates the polarisation of CD4+ T cells towards CD4+IFN-γ+ T cells in active GPA/MPA.

## Figures and Tables

**Table 1:** Patients’ characteristics and comparison between patients with active disease and those with inactive disease.

Variables	Total Patients (N = 20)	Active Patients (N = 10)	Inactive Patients (N = 10)	*p*-Values
Demographic data				
Age (years)	65.5 (50.8–71.0)	71.0 (59.8–75.5)	57.5 (43.3–68.3)	0.023
Female sex (N, (%))	10 (50.0)	5 (50.0)	5 (50.0)	1.000
AAV subtype (N, (%))				
MPA	16 (80.0)	10 (100.0)	6 (60.0)	0.087
GPA	4 (20.0)	0 (0)	4 (40.0)	
ANCA type and positivity (N, (%))				
MPO-ANCA (or P-ANCA)	17 (85.0)	10 (100.0)	7 (70.0)	0.211
PR3-ANCA (or C-ANCA)	3 (15.0)	1 (10.0)	2 (20.0)	1.000
AAV-specific indices				
BVAS	8.0 (4.0–14.8)	14.5 (13.5–16.5)	4.0 (2.75–4.25)	<0.001
SF-36 PCS	52.5 (24.3–67.0)	51.4 (12.2–66.1)	55.6 (38.7–71.2)	0.315
SF-36 MCS	49.3 (42.3–62.3)	46.9 (40.7–66.3)	55.6 (40.7–61.0)	0.739
Laboratory results				
White blood cell count (/mm^3^)	7545.0 (6990.0–10,857.5)	9085.0 (7265.0–13,500.0)	7230.0 (6550.0–9230.0)	0.075
Neutrophil count (/mm^3^)	5570.0 (4440.0–8912.5)	6430.0 (4912.5–10,807.5)	4960.0 (3622.5–6532.5)	0.075
Serum creatinine (mg/dL)	1.1 (0.7–1.8)	1.7 (1.3–2.9)	0.7 (0.7–1.0)	<0.001
ESR (mm/hr)	68.0 (16.0–105.5)	74.0 (41.8–110.3)	32.0 (11.0–84.8)	0.247
CRP (mg/L)	11.0 (1.4–65.6)	25.7 (6.7–97.4)	1.8 (1.2–20.1)	0.105

Values are expressed as a median (25–75 percentile) or N (%). BVAS: Birmingham Vasculitis Activity Score; AAV: ANCA-associated vasculitis; ANCA: antineutrophil cytoplasmic antibody; MPA: microscopic polyangiitis; GPA: granulomatosis with polyangiitis; MPO: myeloperoxidase; P: perinuclear; PR3: proteinase 3; C: cytoplasmic; SF-36: Short-Form 36-Item Health Survey; PCS: physical component summary; MCS: mental component summary; ENT: ear, nose, and throat; ESR: erythrocyte sedimentation rate; CRP: C-reactive protein.

## Data Availability

The data used to support the findings of this study are included within the article and the Supplementary Materials.

## Supplementary Materials

The following supporting information can be downloaded at https://www.mdpi.com/article/10.3390/jcm14248720/s1; Table S1: Detailed clinical manifestations of 20 patients based on individual BVAS Items.
