# Supplementary material for: Rapamycin Modulates the Polarisation of CD4+ T Cells Towards TH1 Cells in Patients with Active Granulomatosis with Polyangiitis and Microscopic Polyangiitis
Source: J Clin Med. 2025 Dec 9;14(24):8720. doi: 10.3390/jcm14248720 (PMC12733731; doi:10.3390/jcm14248720)
Supplement: Supplementary file 1 [file jcm-14-08720-s001.zip › jcm-3938011-supplementary.pdf]

Supplementary Table S1. Detailed clinical manifestations of 20 patients based on individual BVAS Items.

	General	Cutaneous	Mucous membrane/eyes	ENT	Pulmonary	Cardiovascular	Gastrointestinal	Renal	Nervous systemic
Patient 1 (Active)					Infiltrate	Ischemic cardiac pain		Hypertension, Proteinuria>1+, Hematuria≥10RBCs/HPF, Cr 1.41-2.82mg/dL	
Patient 2 (Active)				Paranasal sinus involvement	Infiltrate			Proteinuria>1+, Cr 1.41-2.82mg/dL	
Patient 3 (Active)					Infiltrate	Ischemic cardiac pain		Proteinuria>1+, Cr 1.41-2.82mg/dL	
Patient 4 (Active)	Fever≥ 38°C				Infiltrate			Proteinuria>1+, Hematuria≥10RBCs/HPF	
Patient 5 (Active)					Infiltrate, Massive haemoptysis / alveolar haemorrhage			Proteinuria>1+, Cr 1.41-2.82mg/dL	
Patient 6 (Active)			Conjunctivitis / Blepharitis / Keratitis	Paranasal sinus involvement	Infiltrate			Proteinuria>1+, Hematuria≥10RBCs/HPF	
Patient 7 (Active)				Paranasal sinus involvement	Infiltrate			Hypertension, Proteinuria>1+	
Patient 8 (Active)	Fever≥ 38°C				Pleural effusion / pleurisy, Infiltrate, Massive haemoptysis / alveolar haemorrhage			Hypertension, Proteinuria>1+, Hematuria≥10RBCs/HPF, Cr 2.83-5.64 mg/dL, Rise in Cr > 30% or fall in CCr > 25%	
Patient 9 (Active)	Fever≥ 38°C			Paranasal sinus involvement				Hypertension, Proteinuria>1+, Cr 1.41-2.82mg/dL, Rise in Cr > 30% or fall in CCr > 25%	
Patient 10 (Active)						Valvular heart disease		Proteinuria>1+, Hematuria≥10RBCs/HPF,	

								Cr 2.83-5.64 mg/dL	
Patient 11 (Inactive)						Cardiomyopat hy		Hypertension	
Patient 12 (Inactive)				Paranasal sinus involvement				Hypertension	
Patient 13 (Inactive)					Pleural effusion / pleurisy				
Patient 14 (Inactive)				Paranasal sinus involvement	Nodules or cavities			Hypertension	
Patient 15 (Inactive)				Paranasal sinus involvement, Subglottic stenosis					
Patient 16 (Inactive)	Fever≥ 38°C				Infiltrate				
Patient 17 (Inactive)					Infiltrate				
Patient 18 (Inactive)				Paranasal sinus involvement	Infiltrate			Proteinuria>1+	
Patient 19 (Inactive)					Infiltrate				Sensory peripheral neuropathy
Patient 20 (Inactive)									

BVAS: Birmingham Vasculitis Activity Score; ENT: ear, nose, and throat
